# Ultrasound-Guided Obturator Nerve Block: A Focused Review on Anatomy and Updated Techniques

**DOI:** 10.1155/2017/7023750

**Published:** 2017-02-09

**Authors:** Takayuki Yoshida, Tatsuo Nakamoto, Takahiko Kamibayashi

**Affiliations:** Department of Anesthesiology, Kansai Medical University Hospital, 2-3-1 Shin-machi, Hirakata City, Osaka, Japan

## Abstract

This review outlines the anatomy of the obturator nerve and the indications for obturator nerve block (ONB). Ultrasound-guided ONB techniques and unresolved issues regarding these procedures are also discussed. An ONB is performed to prevent thigh adductor jerk during transurethral resection of bladder tumor, provide analgesia for knee surgery, treat hip pain, and improve persistent hip adductor spasticity. Various ultrasound-guided ONB techniques can be used and can be classified according to whether the approach is distal or proximal. In the distal approach, a transducer is placed at the inguinal crease; the anterior and posterior branches of the nerve are then blocked by two injections of local anesthetic directed toward the interfascial planes where each branch lies. The proximal approach comprises a single injection of local anesthetic into the interfascial plane between the pectineus and obturator externus muscles. Several proximal approaches involving different patient and transducer positions are reported. The proximal approach may be superior for reducing the dose of local anesthetic and providing successful blockade of the obturator nerve, including the hip articular branch, when compared with the distal approach. This hypothesis and any differences between the proximal ONB techniques need to be explored in future studies.

## 1. Introduction

Obturator nerve block (ONB) is commonly performed to prevent sudden thigh adduction during transurethral resection of bladder tumor (TURBT) [1–3], to provide optimal analgesia for knee surgery [4–6], to treat chronic hip pain [7–9], and to improve persistent hip adductor spasticity in patients with paraplegia, multiple sclerosis, or cerebral palsy [10–12]. Labat first described an ONB technique based on surface landmarks in 1922 [13]. Since then, several ONB approaches using surface landmarks with or without nerve stimulation to localize the nerve have been reported [14–16]. During the last decade, ultrasound-guided ONB techniques have gained immense popularity, as have other types of peripheral nerve block. In this review, we describe the anatomy of the obturator nerve, illustrate the ultrasound-guided ONB techniques reported thus far, and identify issues that need to be addressed in the future.

## 2. Anatomy of the Obturator Nerve

**Figure 1:**
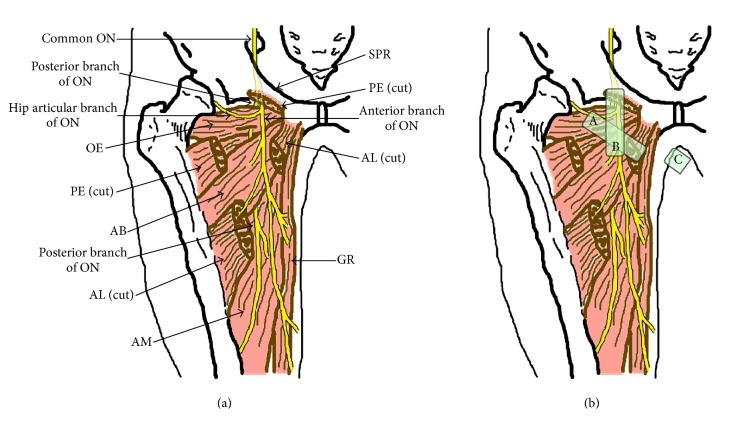
(a) Illustration showing the right-sided adductor muscles and the course of the obturator nerve. (b) Illustration of the positional relationship between the adductor muscles, the obturator nerve, and the transducer during each ultrasound-guided obturator nerve block technique. Large green squares with the letter A or B indicate the foot print position of the transducer. A small green square with the letter C indicates the side position of the transducer. In the distal approach, the transducer is placed at position A. In the proximal approaches reported by Anagnostopoulou et al. [17], Taha [23], and Lin et al. [42], the transducer is tilted cranially at position A to allow visualization of the plane between the pectineus and obturator externus muscles. In the approach used by Akkaya et al. [35], the transducer is placed at position B to allow visualization of the same plane in the sagittal view. Using the approach described by Yoshida et al. [31], the transducer is placed at position C in the lithotomy position to see the plane between the pectineus and obturator externus muscles. ON, obturator nerve; AL, adductor longus muscle; AB, adductor brevis muscle; AM, adductor magnus muscle; PE, pectineus muscle; OE, obturator externus muscle; SPR, superior pubic ramus; GR, gracilis muscle.

The obturator nerve arises from the anterior rami of the second, third, and fourth lumbar nerves. The nerve descends through psoas major and emerges from the medial border of this muscle. The obturator nerve then runs along the lateral wall of the lesser pelvis and extends to the anterior thigh after passing through the obturator canal. During its course, the obturator nerve divides into anterior and posterior branches. In a cadaveric study, bifurcation of these two main branches of the obturator nerve was determined to be intrapelvic (23.22%), within the obturator canal (51.78%), or in the medial thigh (25%) [17]. The anterior and posterior branches of the obturator nerve, or the common obturator nerve, run between the pectineus and obturator externus muscles immediately after the nerve emerges from the obturator canal (Figure 1(a)). Beyond this point, the two branches are usually separated by some of the fibers of the obturator externus muscle. The anterior obturator nerve branch initially passes through the interfascial plane between the pectineus and adductor brevis muscles. Further caudad, it runs between the adductor longus and adductor brevis muscles, innervating the adductor longus, adductor brevis, and gracilis muscles (Figure 1(a)). The anterior branch rarely innervates the pectineus muscle [17]. The posterior obturator nerve branch travels in the fascia between the adductor brevis and adductor magnus muscles (Figure 1(a)). Throughout its course, the nerve usually supplies multiple branches to the adductor magnus and adductor brevis muscles and occasionally innervates the obturator externus and adductor longus muscles as well [17]. The obturator nerve also provides articular branches for the hip and knee joints [17, 18]. The articular branch supplying the hip joint is derived from the common obturator nerve or its branches at different levels in conjunction with the obturator canal [17]. The posterior branch of the obturator nerve supplies terminal branches to the capsule of the knee joint in some individuals [17, 19, 20]. The typical cutaneous distribution of the obturator nerve is described in most textbooks to be the medial side of the thigh and above the medial side of the knee. However, the obturator nerve provides no cutaneous innervation in more than 50% of cases [21].

## 3. Indications

### 3.1. ONB for TURBT

The obturator nerve is situated directly adjacent to the lateral wall of the bladder during TURBT when the irrigation fluid used in this procedure fills the bladder. Any electrical stimulation caused by tumor resection involving the bladder may induce sudden adductor muscle contraction, which may lead to perforation of the bladder accompanied by extravesical spread of the tumor and even injury to the obturator artery [1–3, 22]. Sudden thigh movement and bladder perforation were reported to occur in 40% and 5.7% of patients, respectively, during TURBT involving the lateral wall of the bladder in the absence of ONB [3]. One retrospective study comparing recurrence rates in patients with a bladder tumor on the lateral wall and receiving TURBT with or without ONB demonstrated that an ONB could prolong the mean time to recurrence of the bladder tumor (7.8 months versus 15 months, resp.) [2]. This finding suggests that an ONB can facilitate complete resection of a tumor situated on the lateral wall of the bladder by immobilizing the surgical field. An ONB is essential for performing TURBT safely and effectively.

### 3.2. ONB for Knee Surgery

The knee joint capsule is partially innervated by the obturator nerve [17, 20]. In addition, an ONB is crucial for painless harvesting of the gracilis tendon in anterior cruciate ligament reconstruction because the anterior branch of the obturator nerve innervates the gracilis muscle [17, 23]. Addition of ONB to a femoral nerve block improves analgesia following both total knee replacement [4, 5] and anterior cruciate ligament reconstruction [6, 23].

### 3.3. ONB for Hip Surgery

The hip joint receives sensory innervation from branches of the femoral, obturator, superior gluteal, and sciatic nerves, as well as the nerve to quadratus femoris [18]. Among these, the articular branch of the obturator nerve innervates the anteromedial hip joint capsule. It remains unclear whether an ONB alone can significantly improve the management of acute pain after hip surgery, although one randomized controlled trial demonstrated that a combination of obturator and lateral femoral cutaneous nerve blockade was effective in controlling acute pain after surgery for hip fracture [24].

### 3.4. ONB for Pain Therapy and Hip Adductor Spasticity

Groin and thigh pain frequently arises from the articular branch of the obturator nerve, whereas trochanteric pain arises from the articular branch of the femoral nerve [8]. The nerve responsible for hip joint pain can be detected by a diagnostic nerve block using a local anesthetic. Two case series reports have suggested that percutaneous radiofrequency lesioning or pulsed radiofrequency treatment of the articular branch of the obturator nerve, performed under fluoroscopy guidance, can be an effective alternative treatment in patients with hip joint pain if a diagnostic ONB provides transient pain relief at the hip joint [8, 9].

Persistent hip adductor spasticity is a major complication of spinal cord injury, traumatic brain injury, cerebral palsy, and multiple sclerosis. Hip adductor spasticity causes hip joint deformity, pain, and scissoring of the hips, which prevents maintenance of perineal hygiene, leading to breakdown and infection of the skin in patients requiring long-term care. Some case series have reported that ONB using a neurolytic agent is effective for treatment of hip adductor spasticity in both adult and pediatric patients [10, 11, 25]. Recently, one randomized controlled trial has demonstrated that ONB guided by both ultrasound and electrical simulation using phenol as a treatment for severe hip adductor spasticity in patients requiring long-term care decreases the severity of hip adductor spasticity, improves the hygiene score, and increases the distance between the knees during passive hip abduction [12].

## 4. Evaluation of ONB

As described above, the obturator nerve provides no cutaneous innervation in more than half of individuals; therefore, successful ONB can be achieved despite a lack of sensory block at the medial thigh and/or knee. The success of ONB is evaluated by confirming a decrease in adductor muscle strength using a sphygmomanometer as described by Lang et al. [26]. Using this measurement method, patients are asked to extend both knees fully, dorsiflex both ankles in the supine position, and squeeze a blood pressure cuff (preinflated to 40 mmHg) between their knees by adducting the blocked hip while the nonblocked leg is restrained. The maximal pressure sustained is defined as adductor muscle strength. The adductor magnus muscle is innervated by both the posterior branch of the obturator nerve and the sciatic nerve [27]. Similarly, the femoral nerve also innervates the pectineus muscle [28]. Further, the pectineus muscle is occasionally (in 10%–30% of cases) innervated by the accessory obturator nerve, which arises from the anterior rami of the third and fourth lumbar nerves, descends along the medial border of the psoas major muscle, and passes above the superior pubic ramus [28, 29]. Thus, patients may adduct the hip joint to some extent even if ONB is successful. According to previous studies [30, 31], a decrease in adductor muscle strength of more than 40%–50% has been defined as successful ONB.

## 5. Ultrasound-Guided ONB Techniques

In recent times, ultrasound guidance has been used to improve the success rate and safety profile of peripheral nerve blocks, including ONB. The success rate of landmark-based nerve stimulation-guided ONB in prevention of adductor muscle contraction during TURBT has been reported to be between 84% and 96% [32, 33]. A number of studies have reported that ultrasound-guided ONB is associated with higher success rates of 93%–100% [23, 30, 31, 34–37], although there has been no direct comparison between ultrasound-guided ONB and other techniques. Inadvertent vessel puncture and nerve injury during nerve block procedures would be decreased by correct ultrasound guidance, although unintended obturator vein puncture has been reported even during an ultrasound-guided proximal level ONB [35].

Many ultrasound-guided ONB approaches have been reported and can be classified as distal or proximal.

### 5.1. Distal ONB Approach

**Figure 2:**
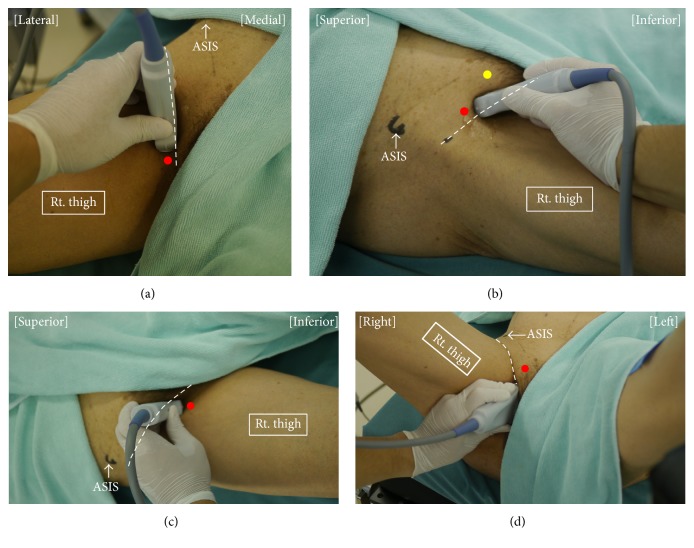
Patient and transducer positions for performance of each ultrasound-guided right-sided obturator nerve block technique. The dotted lines indicate the inguinal crease. (a) The patient is placed in the supine position with his hip slightly abducted and externally rotated. In the distal approach for obturator nerve block, the transducer is placed medial to the femoral vein, along the inguinal crease, perpendicularly to the skin. A needle is inserted from the point indicated by the red circle, in-plane with the transducer, in a medial-to-lateral direction. (b) The transducer is tilted cranially from the position shown in Figure 2(a) to obtain an ultrasound image similar to that in Figure 3(b). A needle is inserted from the point indicated by the yellow circle in an anterior-to-posterior direction using out-of-plane ultrasound guidance in the approach devised by Taha [23]. In the approach used by Lin et al. [42], a needle is inserted from the point indicated by the red circle, in a lateral-to-medial direction, using in-plane ultrasound guidance. (c) The technique described by Akkaya et al. [35] can be performed with the patient in a supine position and his/her leg straight. The transducer is placed in the sagittal plane on the inguinal crease between the femoral vein and the pubic tubercle. A needle is inserted from the point indicated by the red circle, in-plane with the transducer, in an inferior-to-superior direction. (d) In the approach devised by Yoshida et al. [31], the patient is placed in the lithotomy position. The transducer is placed immediately lateral to the perineum on the medial aspect of the thigh along the extended line of the inguinal crease and orientated cephalad. A needle is inserted 2-3 cm cephalad from the anterior side of the transducer (red circle) and advanced in-plane with the transducer in an anterior-to-posterior direction. ASIS, anterior superior iliac spine.

**Figure 3:**
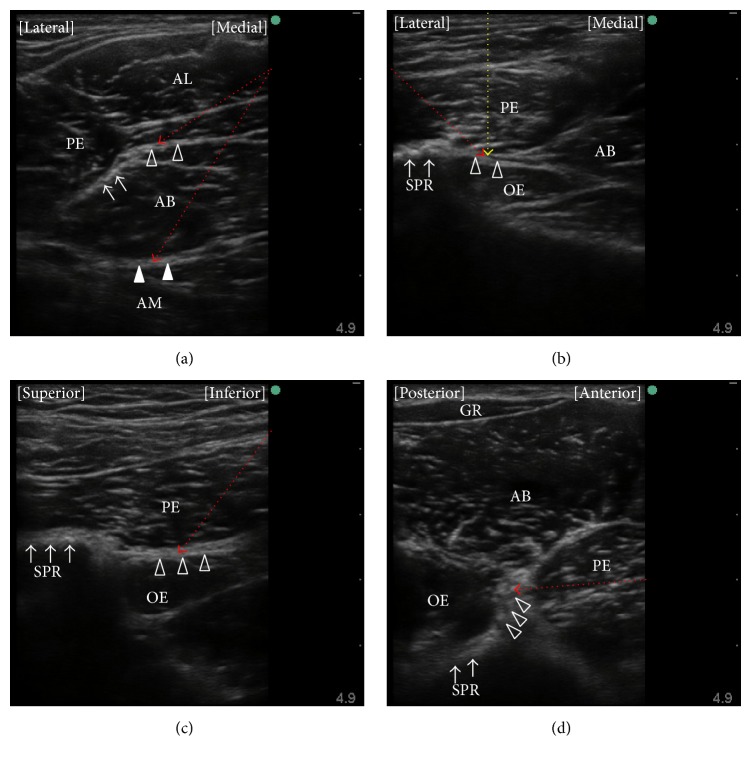
Ultrasound images obtained during obturator nerve block. (a) A preprocedure view of the distal approach, which is obtained with the transducer located as in Figure 2(a). The anterior branch of the obturator nerve is located at the hyperechoic thick fascia between the adductor brevis muscle and the pectineus (arrows) or adductor longus (open triangles) muscles. The posterior branch of the obturator nerve lies within the fascia between the adductor brevis and adductor magnus muscles (closed triangles). A needle is introduced into these fasciae in a medial-to-lateral (red dotted lines) or lateral-to-medial direction under in-plane ultrasound guidance. (b) A preprocedure view obtained with the transducer located as in Figure 2(b). A hyperechoic structure with an acoustic shadow (arrows) represents the superior pubic ramus. The target for injection of local anesthetic is the fascia, which is seen as contiguous with the superior pubic ramus and located between the pectineus and obturator externus muscles (open triangles). In the approach used by Taha [23], a needle is introduced into this plane under out-of-plane ultrasound guidance (yellow dotted line). In the approach used by Lin et al. [42], a needle is inserted into this plane under in-plane ultrasound guidance in a lateral-to-medial direction (red dotted line). (c) A preprocedure view obtained with the transducer located as in Figure 2(c). Hyperechoic thick fascia between the pectineus and obturator externus muscles (open triangles) is seen inferior to a hyperechoic structure with an acoustic shadow, which represents the superior pubic ramus (arrows). A needle is inserted into this fascia under in-plane ultrasound guidance in an inferior-to-superior direction (red dotted line). (d) A preprocedure view obtained with the transducer located as in Figure 2(d). The obturator externus muscle is seen superficial to the superior pubic ramus (arrows) and the pectineus muscle is seen anterior to the obturator externus muscle. A hyperechoic thick fascia between the pectineus and obturator externus muscles (open triangles) is the target plane. As a needle is inserted 3 cm cephalad from the anterior side of the transducer and advanced in-plane with the transducer toward this fascia in this case (red dotted line), the needle-ultrasound beam angles come to be almost perpendicular. AL, adductor longus muscle; AB, adductor brevis muscle; AM, adductor magnus muscle; PE, pectineus muscle; OE, obturator externus muscle; SPR, superior pubic ramus; GR, gracilis muscle.

The distal approach is defined as one in which the anterior and posterior branches of the obturator nerve are blocked separately by two injections of local anesthetic directed toward the interfascial planes where each branch lies. Patients are placed in the supine position with the thigh slightly abducted and externally rotated. An ultrasound transducer is placed at the inguinal crease and perpendicular to the skin (Figures 1(b) and 2(a)) to identify the pectineus, adductor longus, adductor brevis, and adductor magnus muscles (Figure 3(a)). Local anesthetics are injected into the fascia between the pectineus and adductor brevis muscles [30] or between the adductor longus and adductor brevis muscles [38, 39] using in-plane ultrasound guidance to block the anterior branch of the obturator nerve. Subsequently, local anesthetic is injected in the fascia between the adductor brevis and adductor magnus muscles to block the posterior branch of the obturator nerve [30, 38]. Concomitant use of nerve stimulation guidance is recommended when performing ONB using the distal approach because the anatomic variability in the path of the branches of the obturator nerve would be greater at the more distal thigh [30]. The interfascial plane between the adductor brevis and magnus muscles is located relatively deeper. Thus, blockade of the posterior branch requires a steeper angle of needle insertion, which compromises visibility of the needle under ultrasound [40, 41]. ONB at the distal level requires at least two interfascial injections of local anesthetic, so it involves use of a larger volume of local anesthetic than that provided by the single interfascial injection used in the proximal approach, although there has been no research comparing the two approaches in this respect.

Sudden adductor muscle contraction during TURBT might occur even if an ONB using the distal approach is correctly performed because of various patterns of ramification of the obturator nerve when it terminates in the adductor muscles [17]. In other words, an obturator nerve branch, which diverges proximal to the inguinal crease, might not be blocked by the distal ONB approach. Using the distal approach, the local anesthetic injection points are also further away from the bifurcation of the hip joint branch of the obturator nerve when compared with the proximal approach, resulting in less possibility of blockade of the hip joint branch.

### 5.2. Proximal ONB Approach

Taha reported that ultrasound-guided injection of local anesthetic into the interfascial plane between the pectineus and obturator externus muscles successfully produces blockade of both the anterior and posterior branches of the obturator nerve [23]. Yoshida et al. reported that a dye injected into the plane between the pectineus and obturator externus muscles of a cadaver spread into the pelvic cavity through the obturator canal, staining the anterior and posterior branches of the obturator nerve and the common obturator nerve within the obturator canal [31]. Both the anterior and posterior branches of the obturator nerve may run over the obturator externus muscle whereas the posterior branch passes through some fibers of the obturator externus muscle immediately after its emergence from the obturator canal in some cases [17]. Even in these cases, local anesthetics injected into the plane between the pectineus and obturator externus muscles can block both the anterior and posterior branches of the obturator nerve by spreading around these branches and/or the common obturator nerve along the obturator canal, as reported by Yoshida et al. [31]. This retrograde spread of liquid through the obturator canal is key to understanding why the ultrasound-guided proximal approach for ONB, which is provided by a single interfascial injection of local anesthetic, works successfully. Several proximal approaches for ONB have been reported [17, 23, 31, 35, 42], all of which target the plane between the pectineus and obturator externus muscles as the site for injection of local anesthetic but use different patient positions (i.e., supine or lithotomy), transducer locations (i.e., the inguinal crease or medial thigh), modes of needle insertion (i.e., out-of-plane or in-plane), and needle trajectories (i.e., anterior-to-posterior, inferior-to-superior, or lateral-to-medial).

The same patient and transducer positions were used in the reports published by Anagnostopoulou et al. [17], Taha [23], and Lin et al. [42]. These proximal approaches are performed with the patient in the supine position and the hip slightly abducted and externally rotated. The lack of need to change the position of the patient would be the greatest advantage of these techniques when the surgery is also performed in the supine position. A linear ultrasound transducer is first placed at the inguinal crease perpendicular to the skin. Subsequently, the transducer is tilted 40–50 degrees cranially (Figures 1(b) and 2(b)) until a hyperechoic structure deep and lateral to the pectineus muscle, which represents the inferior margin of the superior pubic ramus, is seen (Figure 3(b)). In this view, the target interfascial plane is seen deep in relation to the pectineus muscle separating it from the obturator externus muscle (Figure 3(b)). Using the approach described by Anagnostopoulou et al. [17] and Taha [23], the needle is inserted using out-of-plane ultrasound guidance without nerve stimulation. Out-of-plane ultrasound-guided needle insertion is essentially inferior to an in-plane technique in terms of being able to observe the position of the needle tip in real time [43], although Taha reported a 100% success rate without complication using his approach in 60 patients [23]. The approach devised by Lin et al. [42] entails in-plane lateral-to-medial needle insertion with the same transducer position. In-plane ultrasound guidance allows visualization of the needle tip in real time, which may help to decrease the risk of inadvertent vessel or nerve injury. On the other hand, if the transducer is tilted to a significant degree, it can be difficult to align the needle insertion point with the transducer position and see the needle and target simultaneously under in-plane ultrasound guidance [40].

**Figure 4:**
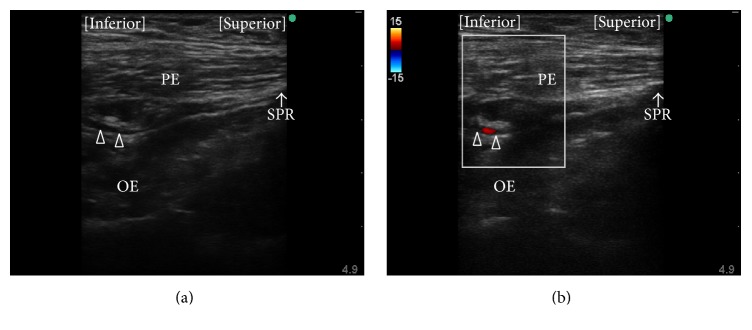
Observation of the obturator artery. (a) Long axis view of a luminal structure (open triangles) is seen at the plane between the pectineus and obturator externus muscles during preprocedure observation using the approach described by Akkaya et al. [35]. (b) The luminal structure was confirmed to be the obturator artery using color flow Doppler. PE, pectineus muscle; OE, obturator externus muscle; SPR, superior pubic ramus.

Akkaya et al. evaluated a further proximal approach in both cadaveric and clinical studies [35]. Using that approach, the patient is placed in the supine position and an ultrasound transducer is introduced in the sagittal plane at the pubic region between the femoral vein and the pubic tubercle (Figures 1(b) and 2(c)) to visualize the superior pubic ramus, the pectineus muscle, and the obturator externus muscle (Figure 3(c)). A block needle is inserted in an inferior-to-superior direction toward the plane between the pectineus and obturator externus muscles under in-plane ultrasound guidance using peripheral nerve stimulation. This approach has a potential advantage contributed by in-plane ultrasound guidance, that is, real-time visualization of the needle tip. However, the relatively steep angle of needle insertion required because of the deep location of the target did not allow good visualization of the needle under in-plane ultrasound guidance. Further, obturator vein puncture occurred in one patient during the clinical application portion of this study [35]. The obturator artery and vein usually descend through the obturator canal from the pelvic cavity; thus, using the approach of Akkaya et al., the long axis of the transducer can be placed parallel to these vessels just as they emerge from the obturator canal [44, 45] (Figure 4). Even slight sliding or tilting of transducer may result in failure to capture an object that exists parallel to the long axis of the transducer under in-plane ultrasound guidance because the width of ultrasound beam is less than 1 mm. We speculate that the inadvertent puncture of an obturator vein in this study occurred because of the positional relationship between the transducer and vessels when this approach is used.

Most recently, Yoshida et al. have described a new approach for proximal level ONB [31]. This approach differs from other ultrasound-guided proximal level approaches in that the interfascial plane between the pectineus and obturator externus muscles is seen from the medial side of the proximal thigh (Figure 1(b)). The transducer cannot be placed at the medial side of the proximal thigh when the patient is in the supine position with the leg straight, so patients are placed either in the lithotomy position or in the supine position with the hip fully flexed and externally rotated. A linear transducer is placed immediately lateral to the perineum on the medial aspect of the thigh along the extended line of the inguinal crease and orientated cephalad (Figure 2(d)). The superior pubic ramus should be identified first, after which the obturator externus muscle can be seen lying superficial to the superior pubic ramus (Figure 3(d)). The pectineus muscle is identified anterior (i.e., on the right hand side of an ultrasound monitor screen) to the obturator externus muscle and the superior pubic ramus (Figure 3(d)). A hyperechoic thick fascia between the pectineus and obturator externus muscles contains the obturator nerve. A needle is inserted a few centimeters (depending on the depth of the target fascia) cephalad from the anterior side of the transducer and advanced in-plane with the transducer toward this fascia. With the patient and transducer positions used in this approach, the needle can be directed almost perpendicularly to the ultrasound beam. Hence, this technique would be theoretically superior for achieving real-time needle visualization when compared with other proximal approaches. A potential disadvantage of this technique is the requirement for the lithotomy position. However, this is not a concern in TURBT because the surgery is performed in the same position.

Nerve stimulation guidance is not always used to perform proximal level ONB procedures because injection of local anesthetic into the interfascial plane between the pectineus and obturator externus muscles, which can be easily identified using ultrasound, provides successful ONB. This interfascial plane is seen as a hyperechoic thick fascia under ultrasound guidance, while the obturator nerve itself can also be seen as a hyperechoic thick structure within this fascia. Therefore, it may be difficult to distinguish the nerve from the interfascial plane under ultrasound guidance alone in some cases. Because an intraneural injection within the perineurium requires a higher injection force than one outside the perineurium, it would be helpful to monitor the injection pressure during ONB, especially in cases without peripheral nerve stimulation, to avoid intrafascicular injection of local anesthetic and the concomitant risk of neurologic complications [46–48].

Technical differences between the above-mentioned ultrasound-guided ONB approaches are summarized in Table 1. Potential advantages and disadvantages of these techniques should be validated in comparative studies in the future.

## 6. Issues to Be Addressed

The superiority of ultrasound-guided ONB techniques over landmark-based ONB techniques has not yet been validated by a prospective comparative trial, although theoretically ultrasound guidance would improve the success rates and safety profiles of peripheral nerve blocks. Various approaches for ultrasound-guided ONB have been reported; however, there has been no study comparing the advantages or disadvantages of these approaches in any respect (e.g., block performance time, success rates, needle visibility under ultrasound, and incidence of complications). The proximal approaches for ONB would be superior to a distal approach for reducing the minimum local anesthetic dose required to achieve blockade and for providing successful blockade of the hip articular branch of the obturator nerve. Nevertheless, this is still a hypothesis that needs to be tested. Further study in a large population would be required to assess the safety of these techniques.

Fluoroscopy guidance was used to perform percutaneous radiofrequency lesioning and pulsed radiofrequency treatment of the obturator nerve for hip joint pain in previous case series reports [8, 9]. These interventions could be performed under ultrasound guidance using a more recent high-performance ultrasound machine with a high-frequency linear transducer, thereby avoiding exposure to radiation.

## 7. Conclusions

Various ultrasound-guided ONB techniques have been reported and can be classified according to whether the approach is distal or proximal. The proximal approach, which comprises a single injection of local anesthetic into the interfascial plane between the pectineus and obturator externus muscles, would be superior for reducing the minimum dose of local anesthetic required and for achieving successful blockade of the obturator nerve, including the hip articular branch, when compared with the distal approach. Nevertheless, this hypothesis should be validated in future studies. The pros and cons of each proximal ONB technique also need to be evaluated in a randomized controlled trial.

## Figures and Tables

**Table 1:** Technical differences in ultrasound-guided obturator nerve block techniques.

	Soong et al. [38] Fujiwara et al. [39] Sinha et al. [30]	Akkaya et al. [35]	Anagnostopoulou et al. [17] Taha [23]	Lin et al. [42]	Yoshida et al. [31]
Ultrasound probe orientation	In-plane	In-plane	Out-of-plane	In-plane	In-plane
Transducer position	Inguinal crease	Pubic region	Inguinal crease	Inguinal crease	Medial thigh
Transducer tilt	Not required	Not required	Required	Required	Not required
Needle-ultrasound beam angle	Small (posterior branch)	Small	NA	Small	Large
Nerve stimulation guidance	Recommended	Recommended	Not needed	Not needed	Not needed
Patient position	Supine	Supine	Supine	Supine	Lithotomy

NA, not applicable.
